# Neuromodulation for Peripheral Nerve Regeneration: Systematic Review of Mechanisms and In Vivo Highlights

**DOI:** 10.3390/biomedicines11041145

**Published:** 2023-04-10

**Authors:** Max Y. Jin, Tristan E. Weaver, Adam Farris, Mayank Gupta, Alaa Abd-Elsayed

**Affiliations:** 1 Department of Anesthesiology, University of Wisconsin-Madison, Madison, WI 53706, USA; 2 Department of Anesthesiology, The Ohio State University Wexner Medical Center, Columbus, OH 43214, USA; 3 Kansas Pain Management & Neuroscience Research Center, Overland Park, KS 66210, USA

**Keywords:** peripheral nerve injury, peripheral nerve regeneration, mechanisms, neuromodulation

## Abstract

While denervation can occur with aging, peripheral nerve injuries are debilitating and often leads to a loss of function and neuropathic pain. Although injured peripheral nerves can regenerate and reinnervate their targets, this process is slow and directionless. There is some evidence supporting the use of neuromodulation to enhance the regeneration of peripheral nerves. This systematic review reported on the underlying mechanisms that allow neuromodulation to aid peripheral nerve regeneration and highlighted important in vivo studies that demonstrate its efficacy. Studies were identified from PubMed (inception through September 2022) and the results were synthesized qualitatively. Included studies were required to contain content related to peripheral nerve regeneration and some form of neuromodulation. Studies reporting in vivo highlights were subject to a risk of bias assessment using the Cochrane Risk of Bias tool. The results of 52 studies indicate that neuromodulation enhances natural peripheral nerve regeneration processes, but still requires other interventions (e.g., conduits) to control the direction of reinnervation. Additional human studies are warranted to verify the applicability of animal studies and to determine how neuromodulation can be optimized for the greatest functional restoration.

## 1. Introduction

Denervation often occurs as a natural part of aging, with studies finding a correlation between age and the number of motor neurons and large-diameter axons [1]. Since muscle fibers require neuromuscular connections, denervation leads to the death and atrophy of the muscles. This results in physical symptoms that include muscle weakness and frailty. Independent of aging, nerve injuries can also result in denervation. Peripheral nerves are most susceptible to injury due to their distribution around the body [2]. Peripheral nerve injuries resulting from trauma or disease are often detrimental and can lead to chronic disabilities [3]. Patients with peripheral nerve injuries frequently suffer from sensorimotor deficits and neuropathic pain [4,5]. In addition to physical symptoms, patients with peripheral nerve injuries may also suffer from psychological symptoms, including depression and decreased self-efficacy [6]. It is estimated that about 2.3% of patients with trauma to the upper or lower extremities experience peripheral nerve injuries [7]. Unlike central nerve denervation, injured peripheral nerves are able to regenerate and reinnervate their targets [8].

It is widely accepted that inactivity can be a cause of denervation [1]. For this reason, exercise is commonly used as an intervention for countering the degeneration of nerves [3]. Pharmaceutically, FK506 (Tacrolimus) is a proven drug that is known to enhance peripheral nerve regeneration [9,10]. Additional pharmacological interventions include B vitamins, exogenous neurotrophic factors, and methylcobalamin [11]. Other common interventions include end-to-end sutures, nerve grafts, conduits, and neurorrhaphy [2]. Surgical suturing is the most effective intervention for peripheral nerve injuries with small gaps, but is ineffective for larger gaps. Large gaps are typically treated by conduits or nerve grafting [12]. Peripheral nerve regeneration is often a slow and directionless process [2]. As a result of this, large gaps decrease the likelihood of nerves reinnervating to the original targets. Additionally, larger gap injuries have reduced Schwann cell support for regenerating axons [13].

Neuromodulation consists of three broad categories of stimulation, all of which provide an alternative for patients with symptoms refractory to other medical interventions [14]. Dorsal column spinal cord stimulation (SCS) and dorsal root ganglion stimulation (DRG-S) both stimulate nerves within the epidural space. A third variety, peripheral nerve stimulation (PNS), targets the nerves outside of the spinal cord that are directly producing the symptoms [15]. All three modalities of neuromodulation have similar indications, although some are more advantageous than others, depending on the patient’s diagnosis. SCS has been the most studied modality and is central to the revolutionary “Gate Control Theory,” which explains the mechanism behind how SCS blocks pain [16]. This theory has since been expanded to explain mechanisms for DRG-S and PNS [17,18,19]. Current indications for SCS include post-laminectomy syndrome, back pain, and numerous other chronic pain conditions [20]. DRG-S has similar indications and has been found to be more efficacious than SCS for focal neuropathies and complex regional pain syndrome (CRPS) [21,22]. Likewise, PNS has indications for neuropathic pain and is best utilized when symptoms can be traced to a specific nerve [23].

Specific symptoms treated by neuromodulation include pain associated with disease or trauma, urological conditions, and other functional disorders [24]. Common questionnaires to assess these symptoms include the Visual Analogue Scale (VAS) and the Numeric Rating Scale (NRS) for pain, and the Short-Form Health Survey (SF-36) for physical function. Currently, neuromodulation is reserved for when symptoms are refractory to other treatment modalities [25]. Prior to intervention, patients first undergo a diagnostic nerve block to confirm that the correct nerve is targeted and that the patient experiences at least a 50% reduction in pain [14]. This is followed by a trial period where temporary electrodes are implanted to ensure that the patient’s symptoms respond to the neuromodulation intervention. Only after confirmation will a permanent system be implanted.

Neuromodulation is an intervention that can be applied “off-label” to enhance peripheral nerve regeneration. The diagnostic basis for neuromodulation is the same for surgical interventions and includes an electrophysiological examination of motor nerve conduction and the physical symptoms supporting nerve injury (e.g., numbness, weakness). Specifically, there is some evidence that PNS/electrical stimulation (ES) is effective [26,27]. Jo et al. demonstrated in a comparative study on mice that ES is equally as efficacious as FK506 (Tacrolimus) treatment [28]. Despite this evidence, the exact mechanism of action is relatively unknown.

This review will evaluate all available literature that includes any data regarding neuromodulation and peripheral nerve regeneration. The purpose of this review is to formulate the underlying mechanisms by which neuromodulation is able to impact peripheral regeneration and highlight in vivo data that reports on its utility.

## 2. Materials and Methods

### 2.1. Search Strategy

This study abided by the Preferred Reporting Items for Systematic Reviews and Meta-Analysis (PRISMA) guidelines and was registered in the International Prospective Register of Systematic Reviews (PROSPERO ID CRD42023394553) [29]. A PubMed search was conducted for articles from inception through to September 2022. Our search syntax consisted of Boolean operators and broad MeSH terms such as “Spinal Cord Stimulation,” “Electric Stimulation,” “Peripheral Nerves/injuries,” and “Nerve Regeneration.” Other keywords search for in the titles/abstracts included terms and synonyms for “Peripheral Nerve Stimulation,” “Dorsal Root Ganglion Stimulation,” and “neuromodulation.” All articles searched were filtered for English language only. This search strategy was verified by a senior academic librarian (Leslie Christensen). The exact search syntax utilized with the number of results can be viewed in Supplementary Table S1.

### 2.2. Study Selection

Studies included could be either in vitro or in vivo. In vivo studies could be human or animal studies. Other inclusion criteria comprised the use of any modality of neuromodulation (e.g., SCS, DRG-S, PNS/ES) to promote the regeneration of peripheral nerves. The regeneration of peripheral nerves could be evidenced by functional recovery, evidence of remyelination, changes in neural plasticity (i.e., sprouting or rerouting), or axon regeneration. Articles that were excluded included those that were non-peer-reviewed, non-English language, or where nerves were not directly stimulated (e.g., stimulation of muscles). Two authors (M.Y.J. and T.E.W.) independently selected articles, with a third author (A.A.-E.) serving as the tiebreaker.

### 2.3. Bias Assessment

The assessment of bias was conducted only on studies that report in vivo data. Two authors (M.Y.J. and A.F.) assessed biases, while another author (A.A.-E) resolved any discrepancies. The Cochrane Risk of Bias tool was utilized to assess randomized controlled trials (RCTs) [30]. The biases assessed by this validated tool were selection, performance, detection, attrition, reporting, and other biases. Each domain of bias was evaluated as low risk, high risk, or unclear risk.

## 3. Results

### 3.1. Search Results

**Figure 1:**
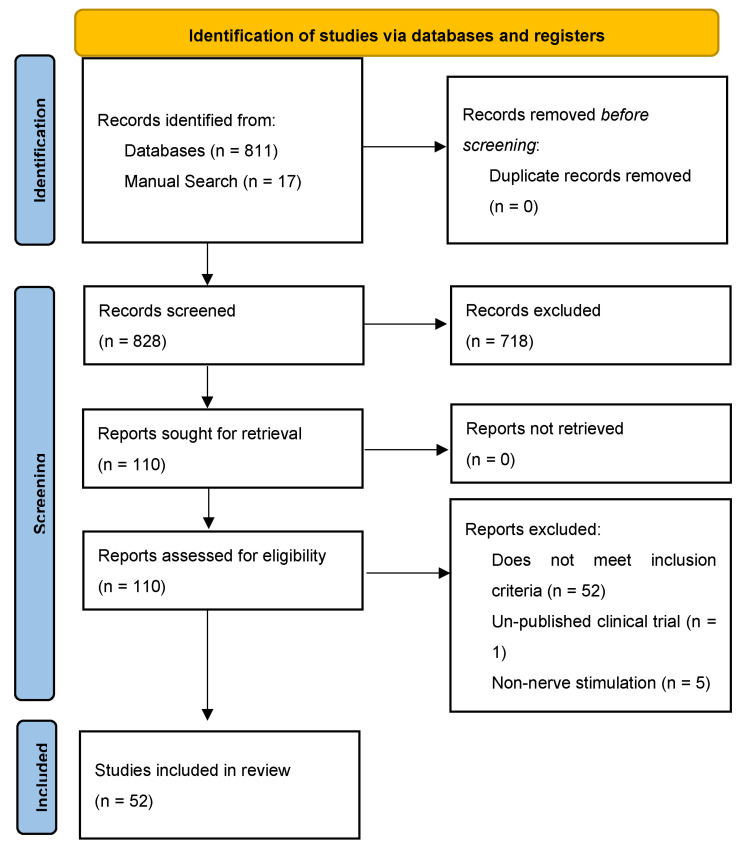
PRISMA Flow Diagram [80].

Our search strategy yielded 811 studies. We manually identified 17 articles on PubMed to provide evidence for additional mechanisms that were not reported in the articles found through the search strategy. After a duplicate and independent screening of titles and abstracts, we found an abundance of articles that satisfied our inclusion criteria. For this reason, we decided to further filter the results to only include articles from the last 10 years (2012–2022) in order to provide the most up-to-date information. In addition, only RCTs (regardless of date) were included for highlighting in vivo outcomes, to provide the highest level of evidence available. Animal RCTs were included to examine other independent variables that have not yet been analyzed in humans. A total of 52 articles were selected for inclusion, with 45 studies [8,11,26,31,32,33,34,35,36,37,38,39,40,41,42,43,44,45,46,47,48,49,50,51,52,53,54,55,56,57,58,59,60,61,62,63,64,65,66,67,68,69,70,71,72] on mechanisms and seven RCTs [73,74,75,76,77,78,79]. A complete description of the search results is presented in Figure 1.

### 3.2. Mechanisms

#### 3.2.1. Biological Mechanisms

**Figure 2:**
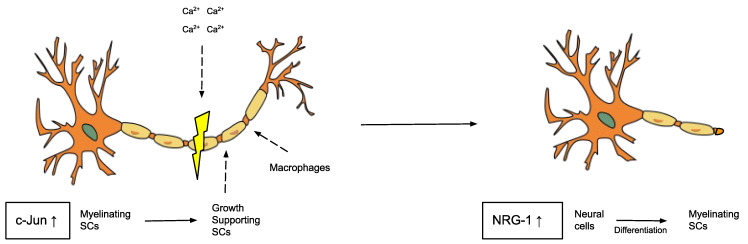
Wallerian Degeneration. NRG-1—neuregulin-1; SC—Schwann cell.

Prior to the discussion of possible mechanisms through which neuromodulation is able to aid peripheral nerve regeneration, it is important to first understand naturally occurring nerve regeneration mechanisms. After a peripheral nerve is severed or denervated, the nerves first undergo Wallerian degeneration (Figure 2, Table 1). Calcium flows into the site of injury, with lower concentrations of calcium flowing around the distal axons [8,33]. The calcium influx is required for axon regeneration to begin, triggering the degradation of the axons with myelin by Schwann cells (SCs) and macrophages. Prior to this process, SCs first transition from myelinating SCs to growth supporting SCs [59]. The transcriptional factor c-Jun has been shown to be upregulated in SCs of injured nerves as a key factor in promoting the regeneration process [60]. Debris of severed axons distal to the site of the injury is subsequently cleared by macrophages recruited through the binding of chemokine C-C motif ligand 2 (CCL2) onto C-C chemokine receptor type 2 (CCR2) [34,37,53]. Wallerian degeneration also initiates the expression of neuregulin-1 (NRG-1) in SCs to stimulate the differentiation of additional SCs and remyelination [51,61].

**Figure 3:**
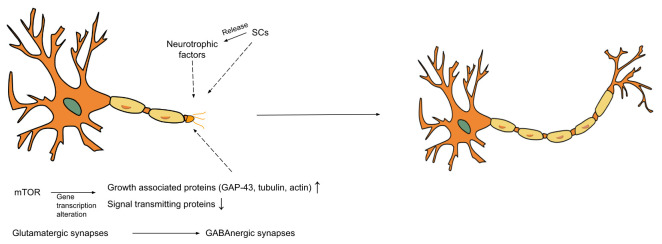
Regeneration Summary. mTOR—rapamycin; SC—Schwann cell.

Following this degeneration process, several growth-associated genes that transcribe proteins including GAP-43, tubulin, and actin are upregulated in proximal axons, while genes that transcribe signal transmitting proteins are downregulated (Figure 3, Table 1) [8,36]. The alteration of gene transcription is achieved through the synthesis of the protein rapamycin (mTOR). This protein is able to control the mRNA translation localized in the axons. Within five days of denervation, SCs initiate the expression of neurotrophic factors that are crucial for nerve regeneration [8,38]. Axon sprouts grow in a mostly directionless manner out of the proximal stump [8]. Polysialic acid is a key molecule that contributes to the direction of growing axons. It is required for the reinnervation of the correct muscle [62]. During this process, fast excitatory glutamatergic synapses are replaced by slower GABAnergic depolarizing signals in order to optimize regeneration [52,53]. Additional processes include the upregulation of the NRG-1 isoform glial growth factor (GGF), select microRNAs (miR-3099 and miR-sc3) and long noncoding RNAs (Ngrl1) to promote SC proliferation and migration [48,49,54,58]. The biological peripheral nerve regeneration process is largely time-dependent, with the the growth factors of SCs peaking at 15 days and returning to baseline by 35 days [35]. Other microRNAs (miR-9, miR-182, miR-340, miR-sc8, miR-1, and miR-129) and long noncoding RNAs (Arrl1, Loc680254, and TNXA-PS1) are upregulated early, near the onset of injury for the inhibition of SC migration and clearance of debris [42,46,55,56,57,63,64,65,66]. The impact of microRNAs and long noncoding RNAs on peripheral nerve regeneration is still a relatively new concept, so more research is needed to determine the exact mechanisms they undergo.

#### 3.2.2. ES Mechanisms

So how is neuromodulation able to aid in the regeneration and reinnervation of peripheral nerves? First, it is important to note that ES is the only modality that has been extensively investigated in present studies, so the majority of our discussion will be on ES. Second, ES has been found to result in the misdirection of regenerated axons, so its utility exists only when the nerve is not completely severed (specifically, endoneurial tubes need to be intact) or when other interventions such as conduits are employed [32,36,61].

ES targets the neurons in regenerating axons and works to increase the activity [31]. The microenvironment can be optimized for regeneration by ES through the increase in calcium levels. A calcium-rich environment is required for the initiation of axon outgrowth, and experiments have demonstrated that ES promotes the rise of intracellular calcium, possibly due to the alteration of voltage-gated calcium channels [33,41]. By increasing the neuronal calcium levels, ES may also indirectly stimulate the increased expression of brain-derived neurotrophic factor (BDNF), as well as other neurotrophins and their receptors (e.g., Tropomyosin receptor kinase B [TrkB]) [31,32,35,43,47,67,68]. It has been discovered that the effects of ES are significantly reduced when the effect of BDNF is blocked by antibodies or when the BDNF gene is knocked out in mice [31,43]. The increase in BDNF and TrkB expression may also be due to the impact of ES on androgens [31]. Both molecules’ expression can be stimulated by androgens. ES mimics the effects of exercise, which has been shown to modulate testosterone [31,69]. This mechanism of action is supported by studies where the presence of an androgen inhibitor (flutamide) or the deletion of genomic androgen receptor signaling resulted in no significant regeneration effect by ES [39,40]. ES can also aid in the upregulation of growth-associated genes through the increased activation of mTOR [70]. The increased production of SCs, the molecules that secrete neurotrophic factors, may provide another explanation for the role of ES in peripheral nerve regeneration [33]. One possible explanation for the increase in SCs is that ES upregulates NRG-1 expression [71]. There has been evidence that ES can increase NRG-1 expression in muscle cells, but the effect of ES on neural cells has not been examined. In an in vivo study on Sprague–Dawley rats, Gu et al. found that low-frequency ES had the ability to increase the proliferation and differentiation of peripheral blood stem cells into SCs through alteration of the extracellular signal-regulated kinase (ERK) signaling pathway [50]. The alteration of the ERK signaling pathway by ES may also upregulate select microRNAs that promote axon outgrowth; however, there are limited studies that have investigated this [72]. There has been no evidence of ES modulating the expression of long noncoding RNAs.

Recent research has revealed other possible mechanisms. English et al. reported in a mice study that the repression of asparagine endopeptidase (AEP) may be part of the ES mechanism [44]. AEP acts on two molecules (Tau and amyloid precursor protein) that antagonize nerve regeneration after peripheral nerve injury. When mice were knock-out for the AEP gene, they experienced greater axon outgrowth than the wild-type. After one hour of ES to the AEP knock-out group, no additional outgrowth was observed. It was proposed that the BDNF/TrkB signaling induced by ES could impair the activity of AEP. The efficacy of ES to aid accurate reinnervation still depends on the expression of polysialic acid by the axotomized motor neurons [35,62]. Mice studies have found that the accuracy of peripheral nerve regeneration after ES was lost when the acid was blocked or not present [35].

#### 3.2.3. Other Neuromodulation Mechanisms

As previously mentioned, other neuromodulation interventions, including SCS and DRG-S, have limited literature to support their use in peripheral nerve regeneration. However, there may be promise in those interventions. In rat studies, it has been found that SCS is able to reduce harmful apoptosis during Wallerian degeneration that slows regeneration [11,45]. SCS in a mouse model also demonstrated significantly greater expression of polysialic acid, indicating the possible ability of SCS to improve the accuracy of reinnervation [35]. Additionally, it has been proposed that DRG-S is able to reduce DRG excitability, altering DRG neuronal activity [11]. However, this has only been shown to repress the neuropathic pain induced by peripheral nerve injuries, with no evidence that it aids the regeneration process. The mechanisms of neuromodulation interventions on peripheral nerve regeneration are summarized in Table 2.

### 3.3. In Vivo Highlights

In total, seven RCTs were found to provide evidence of efficacy, with four animal studies [73,74,75,76] and three human studies [77,78,79]. A summary of the findings is reported in Table 3. All studies utilized ES to supplement surgical intervention or conduit repair. Of the four animal studies, Senger et al. was the only one that investigated conditioning ES [75]. In their study of 122 rats that underwent tibial nerve transection, it was found that 60 min conditioning (i.e., seven days prior to surgery) ES alone was significantly more beneficial than post-operational ES, a combination of conditioning and post-operational ES, and no ES. The conditioning ES group was found to have the greatest length and number of axons after one week as well as the greatest sensory and motor recovery after six weeks. Roh et al. also conducted a study on rats after tibial nerve transection [73]. In 39 rats, it was found that the 10 min ES group had the greatest axon outgrowth at two weeks, while the 60 min ES group still had greater axon outgrowth than the control. At 52 weeks, both ES groups demonstrated significantly higher tibial nerve function than the control group, despite the control group having a significantly greater number of myelinated axons than the 10 min ES group.

Calvey et al. also conducted a study on rats, but investigated the injury of the sciatic nerve rather than the tibial [76]. In 41 rats, the 10 min ES group outperformed all other groups (60 min ES, no ES, isograft) in sciatic nerve function and extensor potential thrust at week 12. Although the isograft group was found to have the highest number of nerve fibers at the midline of the conduit, both ES groups had the greatest number of nerve fibers at 2 mm distal to the repair conduit. Sayanagi et al. was the only study that examined peripheral nerve regeneration in mice rather than rats [74]. In the 99 mice that underwent sciatic nerve transection, both the 10 min and 60 min ES groups outperformed the control in terms of labeled motoneurons and myelinated axons at 15 days. Additionally, at 56 days, both groups outperformed the control on the grid walking test and on mechanical sensitivity. However, no significant difference in cold sensitivity was reported in any of the groups.

All three clinical studies on humans also demonstrated the benefits of ES on peripheral nerve regeneration. In two separate studies, Power et al. and Gordon et al. examined participants recovering from cubital tunnel syndrome (n = 31) and carpal tunnel syndrome (n = 21), respectively [77,79]. Both studies found 60 min ES to be beneficial in improving functional and physiological outcomes compared to surgical intervention alone. Gordon et al. even reported in their study that the MUNE (motor unit number estimate) of the 60 min ES group was similar to that of a healthy hand [79]. Lastly, Wong et al. reported on 36 patients after complete digital nerve transection [78]. Their study found that 60 min ES restored CDT (quantitative cold detection) and DASH (disabilities of the arm, shoulder, and hand) scores, indicating improved temperature sensitivity and function. In addition, it was reported that the recovery of the sham stimulation group plateaued at 3–4 months.

### 3.4. Bias Assessment

**Figure 4:**
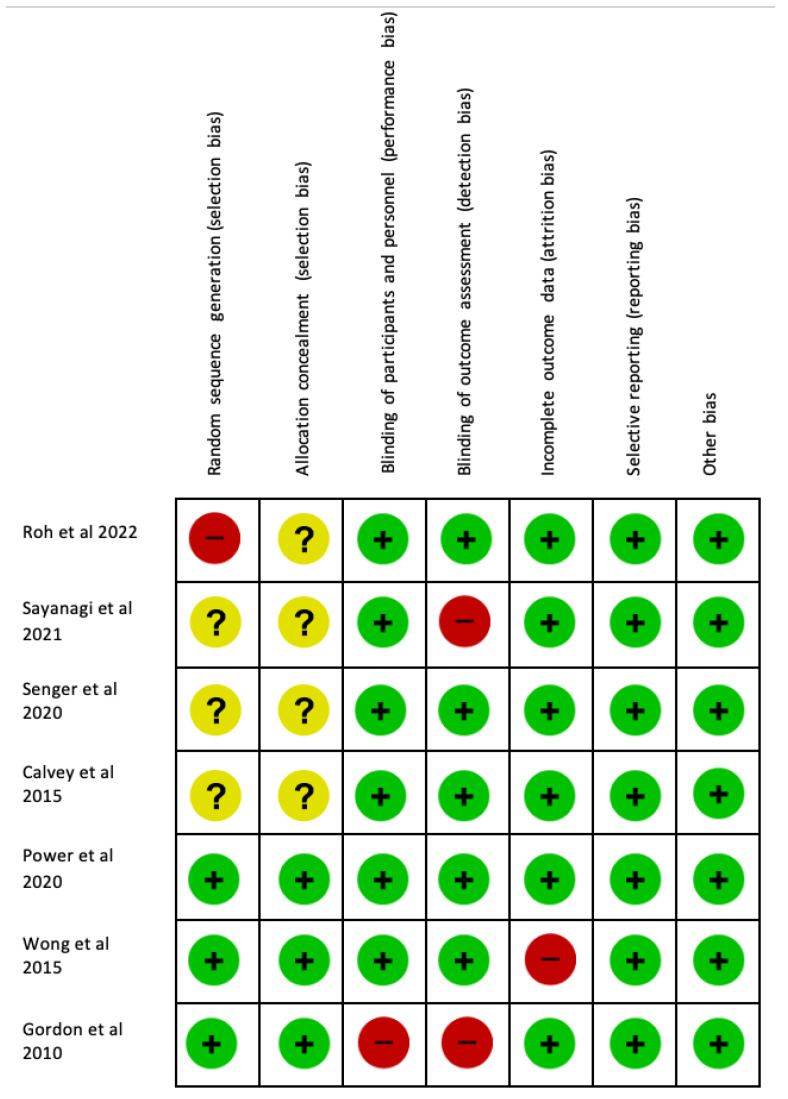
Cochrane Risk of Bias Assessment [73,74,75,76,77,78,79].

The assessment of bias using the Cochrane Risk of Bias assessment tool is summarized in Figure 4. All animal studies demonstrated an unclear [74,75,76] or high risk [73] of selection bias due to the lack of discussion of randomization protocols or allocation by the clinician. Additionally, one animal study demonstrated a high risk of detection bias due to the lack of blinding of the assessors [74]. A low risk of bias was found for the remaining domains in animal studies. For human studies, one study demonstrated low risk for all domains of bias [77]. Another study was low risk for all domains except attrition bias where more than 10% of participants were lost at follow-up with no explanation [78]. The final study demonstrated low risk for all domains except for performance and detection because no blinding was utilized [79].

## 4. Discussion

This review synthesized the current understanding of neuromodulation mechanisms responsible for the enhancement of peripheral nerve regeneration. Results show that ES can speed up the regeneration process by increasing the influx of calcium, which stimulates the expression of neurotrophic factors and their receptors [31,32,33,35,41,43,47,67,68]. Additionally, the use of ES has demonstrated success in increasing the expression of androgens, NRG-1, and mTOR, as well as impairing the activity of AEP [31,44,70,71]. Finally, ES can stimulate the proliferation and differentiation of SCs [33,50]. Evidence for SCS and DRG-S is much more limited but shows promise in reducing harmful apoptosis and altering DRG neuronal activity, respectively [11,45]. SCS can also increase the expression of polysialic acid [35]. The poor functional outcomes of peripheral nerve regeneration are largely attributed to chronic denervation (due to delayed repair), and a lack of direction in the regenerating axons [81]. Neuromodulation is able to accelerate the regeneration process, but remains unable to control the direction of regenerating axons to reinnervate the correct targets [82].

An important distinction to make is when neuromodulation should be implemented, whether it be pre-, peri-, or post-operative. For most chronic pain conditions, neuromodulation is utilized as part of the rehabilitative treatment for symptoms that were unable to be treated by other interventions [19]. However, due to the consensus that current treatments for peripheral nerve injuries cannot adequately restore function, it may be wise to consider the implementation of neuromodulation as part of the surgical treatment [36]. Perioperative neuromodulation avoids multiple surgeries to obtain the relief of symptoms, as leads can be implanted (and even explanted in the case of temporary sessions) during the operation itself. Additionally, conditioning (i.e., preoperative) stimulation should be considered due to the time sensitive nature of peripheral nerve regeneration. Delayed surgical intervention is often the norm in humans because of the difficulty in diagnosing peripheral nerve injuries, so non-invasive neuromodulation technologies such Transcutaneous Electrical Nerve Stimulation (TENS) have possible utility as a means to prepare for surgery [26,83]. Animal models have shown that conditioning stimulation can optimize the microenvironment of the injured nerve to promote greater efficacy of surgical interventions [84].

There are several limitations to address for this review. With regard to limitations in the reviewed studies, four [73,74,75,76] of the seven RCTs highlighting in vivo outcomes were animal studies that may not be directly translatable to humans. Similarly, most of the evidence supporting the proposed mechanisms were also from animal studies. Additionally, specific stimulation settings were not reported in the majority of the studies on regeneration mechanisms. Studies frequently referred to the stimulation settings as simply “low-frequency” without any further details. There was a high level of heterogeneity in the injury models leading to variations in implantation sites and stimulation settings. Injuries were also treated in different ways, with some studies using conduits and others using surgical suturing. Additional limitations were related to our review processes. Due to the heterogeneity in injuries and other interventions applied, no GRADE (Grading of Recommendations, Assessment, Development, and Evaluations) assessment could be completed to assess the quality of the evidence. Similarly, the heterogeneity of studies resulted in an inability to pool results for a quantitative analysis of in vivo outcomes. Another limitation of our review process was that we excluded studies on mechanisms published prior to 2012 in an effort to report the most current evidence. We ended up having to include three studies [61,67,69] published prior to 2012 post-hoc to supplement the results of more recent publications.

## 5. Importance of Animal Models

Animal studies remain important in the study of peripheral nerve regeneration because they come with the ability to examine unique independent variables to optimize ES treatment. In our review, the human clinical studies only utilized 60 min ES, while the animal studies altered the duration of ES and even examined differences in the timing of the application of ES. Only the 60 min ES protocol was utilized for humans because it has been studied the most and has the greatest evidence of being effective [79]. Clinicians conducting human clinical studies must consider what is good for the patient prior to determining what is good for the study itself [85]. Experiments on animals can explore novel ES and other neuromodulation protocols to provide evidence for efficacy in humans. Only when there is ample evidence supporting its efficacy, can human studies on the same protocol be conducted. However, it is important to view these results with caution because animal models are not perfect. Complete transection models are difficult to translate to humans because most nerve injuries experienced by humans, other than amputations, are partial lesions [86]. Other injury models come with the difficulty of inducing the same extent of injury to all animals used.

## 6. Future Directions

Future studies should examine whether the results from animal models are translatable to humans, and investigate how ES can be optimized. Current human studies have demonstrated similar successes as animal models, but the mechanisms of action require further evaluation. Clinicians may need to inquire more about the phenomenon of conditioning ES as current studies focus on peri-operative or post-operative ES. Due to the current challenge of the complete restoration of function after peripheral nerve injuries, and the roles of spinal cord and dorsal root ganglion neurons in regeneration, other neuromodulation interventions such as SCS and DRG-S warrant further evaluation. Additionally, future studies should report data on lead contact patterns as the efficacy may vary depending on which contact pattern is utilized [87].

## 7. Conclusions

ES is effective in the enhancement of peripheral nerve regeneration. Other neuromodulation interventions show promise but require further research to support their efficacy. Neuromodulation works by supporting existing biological mechanisms to accelerate regeneration. While neuromodulation can accelerate this time sensitive process, it is still unable to control the direction of regeneration. Other interventions that can guide the regenerating axons (e.g., conduit) are still required in order to obtain optimal functional outcomes with neuromodulation.

## Figures and Tables

**Table 1:** Summary of Biological Mechanisms.

Wallerian Degeneration
- Calcium influx to site of injury [8,33] ○ Trigger degradation of axons with myelin - c-Jun upregulated in SCs of injured nerves [59,60] ○ Myelinating SCs → growth supporting SCs - Debris clearance by macrophages [34,37,53] - Upregulation of select microRNAs * (miR-9, miR-182, miR-340, miR-sc8, miR-1, miR-129) and long noncoding RNAs * (Arrl1, Loc680254, TNXA-PS1) [55,56,57,63,64,65,66] ○ Inhibit SC migration - Results in increased expression of NRG-1 in SCs [51,61] ○ Neural cells → Myelinating SCs
**Regeneration**
- Alteration of gene transcription through mTOR synthesis [8,36] ○ Upregulation of growth-associated genes ○ Downregulation of signal transmitting proteins - SC expression of neurotrophic factors necessary for regeneration [8,38] - Presence of polysialic acid to control direction of regenerating axons [62] - Fast excitatory glutamatergic synapses → slower GABAnergic depolarizing signals [52,53] - Upregulation of GGF, and select microRNAs * (miR-3099, miR-sc3) and long noncoding RNAs * (Ngrl1) [48,49,54,58] ○ Promote SC proliferation and migration

* Requires further investigation to understand specific mechanisms of action. SC—Schwann cell; NRG-1—neuregulin-1; mTOR—rapamycin; GGF—glial growth factor.

**Table 2:** Summary of Neuromodulation Mechanisms.

Intervention	Key Mechanisms
ES	- Alteration of voltage-gated calcium channels to promote intracellular calcium [33,41] - Increase expression of neurotrophic factors and receptors [31,32,35,43,47,67,68] - Increase expression of androgens [31] - Increase activation of mTOR [70] - Upregulate expression of NRG-1 [71] - Alter ERK signaling pathway for increased proliferation and differentiation of Schwann cells [50] - Impair activity of AEP [44]
SCS	- Reduce harmful apoptosis during Wallerian degeneration [11,45] - Increase expression of polysialic acid [35]
DRG-S	- Reduce DRG excitability [11] - Alter DRG neuronal activity [11]

ES—Electrical stimulation; mTOR—rapamycin; NRG-1—neuregulin-1; ERK—extracellular signal-regulated kinase; AEP—Asparagine endopeptidase; SCS—Spinal cord stimulation; DRG-S—Dorsal root ganglion stimulation.

**Table 3:** Summary of In-Vivo Highlights from RCTs.

Author	Study Population	Injury Type	Stimulation Settings (Location)	Arms	Outcomes
Roh et al., 2022 [73]	39 rats	Tibial nerve transection ^Ψ^	100 µs, 0.5 mA, 16 Hz (2 mm proximal to the cut/repair site and proximal to injury site)	10 min ES (n = 13) vs. 60 min ES (n = 13) vs. no ES control (n = 13)	At 2 weeks, the 10 min ES group had greater axon outgrowth than the 60 min ES group which had greater outgrowth than the no ES groups. At 52 days, 10 min and 60 min ES groups both had similar TFI scores that were significantly greater than the control. The control group had a significantly greater number of myelinated axons than the 10 min ES group.
Sayanagi et al., 2021 [74]	99 mice	Sciatic nerve transection ^Ψ^	0.5 mA, 16 Hz (2 mm proximal to the repair site)	10 min ES (n = 33) vs. 60 min ES (n = 33) vs. no ES control (n = 33)	At 15 days, both ES groups had a significantly greater number of labeled motoneurons and myelinated axons than the control. At 56 days, both ES groups also outperformed the control on the grid walking test and on mechanical sensitivity. No significant differences in cold sensitivity were found for any of the groups.
Senger et al., 2020 [75]	122 rats	Tibial nerve transection ^Ψ^	0.1 ms, 20 Hz (tibial nerve)	CES ^Ω^ (n = NR) vs. PES vs. CES + PES vs. control	At 1 week, CES group had significantly greater length and number of axons regenerated than all other groups. At 6 weeks, CES significantly outperformed all groups in sensory and motor recovery.
Calvey et al., 2015 [76]	41 rats	10-mm Sciatic nerve injury ^±^	24 mV, ∼1.5 μA (proximal and distal nerve segments)	10 min ES (n = 10) vs. 60 min ES (n = 11) vs. no ES control (n = 10) vs. Isograft control (n = 10)	SFI and extensor potential thrust at week 12 were greatest in 10 min ES group. The number of nerve fibers at the midline of the conduit was greatest in isograft control and at 2 mm distal to the repair conduit was equally greatest for both ES groups and isograft control
Power et al., 2020 [77]	31 humans	Cubital tunnel syndrome *	<30 V, 0.1 ms, 20 Hz (ulnar nerve proximal to the site of compression)	60 min ES (n = 20) vs. no ES control (n = 11)	At 3 years, the ES group demonstrated significantly higher MUNE and key pinch strength than control. Other functional and physiological outcomes were also significantly improved in the ES group compared to the control.
Wong et al., 2015 [78]	36 humans	Complete digital nerve transection ^Ψ^	<30 V, 0.1–0.4 ms, 20 Hz (proximal to surgical site)	60 min ES (n = 16) vs. sham (n = 16)	ES group reported greater restoration of CDT and DASH scores. Based on the MRC Modified Highet Scale, 86.7% of the ES group experienced normal recovery in tactile discrimination and pressure detection as compared to 43.8% of the sham group. Sham group recovery plateaued at 3–4 months.
Gordon et al., 2010 [79]	21 humans	Carpal tunnel syndrome *	4–6 V, 0.1–0.8 ms, 20 Hz (medial nerve above the site of compression)	60 min ES (n = 11) vs. no ES control (n = 10)	MUNE, terminal motor latency, and sensory nerve conduction significantly improved for the ES group but not control. MUNE of the ES group was not statistically different from a healthy hand.

^Ψ^ All patients underwent epineurial nerve repair surgery first; ^Ω^ CES—conditioning 60 min ES 7 days prior to surgery; PES—60 min postoperative ES; ^±^ all rats underwent conduit repair first; * all patients underwent carpal or cubital tunnel release surgery first; TFI—tibial functional index; SFI—sciatic functional index; MUNE—motor unit number estimate; CDT—quantitative cold detection threshold; DASH—disabilities of the arm, shoulder, and hand; MRC Modified Highet Scale—Dellon’s modification of the British Medical Research Council Highet scale for grading sensory recovery.

## Data Availability

Data are available upon request to the corresponding author.

## Supplementary Materials

The following supporting information can be downloaded at: https://www.mdpi.com/article/10.3390/biomedicines11041145/s1, Table S1: PubMed Search Syntax.
