# Supplementary material for: Neuromodulation for Peripheral Nerve Regeneration: Systematic Review of Mechanisms and In Vivo Highlights
Source: Biomedicines. 2023 Apr 10;11(4):1145. doi: 10.3390/biomedicines11041145 (PMC10135453; doi:10.3390/biomedicines11041145)
Supplement: Supplementary file 1 [file biomedicines-11-01145-s001.zip › biomedicines-2246447-supplementary.pdf]

Table S1. PubMed Search Syntax.

**Search Syntax**	**Results**
("Electric Stimulation"[mesh] OR "Electric Stimulation Therapy"[mesh] OR "Spinal Cord Stimulation"[mesh] OR (electrostimulat*[tiab] OR electromodulat*[tiab] OR neurostimulat*[tiab] OR neuromodulat*[tiab] OR electro-stimulat*[tiab] OR electro-modulat*[tiab] OR neuro-stimulat*[tiab] OR neuro-modulat*[tiab] OR ((electric[tiab] OR electrical[tiab] OR spinal*[tiab] OR dorsal-column*[tiab] OR dorsal-root*[tiab] OR peripheral-nerve*[tiab]) AND (stimulation*[tiab] OR stimulator*[tiab] OR modulation*[tiab] OR modulator*[tiab])))) <br>AND ("Peripheral Nerves/injuries"[mesh] OR "Peripheral Nerve Injuries"[mesh] OR ((peripheral-nerve*[tiab] OR epineurium*[tiab] OR endoneurium*[tiab]) AND (injur*[tw] OR injuries[sh]))) <br>AND ("Nerve Regeneration"[mesh] OR ((nerve*[tiab] OR nerve-tissue[tiab] OR neural-tissue*[tiab] OR nervous-tissue*[tiab]) AND (regenerat*[tiab]))) <br>AND English[filter]	811
